# Estimation of *Candida albicans* ABC Transporter Behavior in Real-Time via Fluorescence

**DOI:** 10.3389/fmicb.2015.01382

**Published:** 2015-12-09

**Authors:** Joanna Szczepaniak, Marcin Łukaszewicz, Anna Krasowska

**Affiliations:** Faculty of Biotechnology, University of WroclawWroclaw, Poland

**Keywords:** diS-C_3_(3) fluorescence, protein localization imaging, kinetics of eﬄux pump activity, *Candida albicans*, CDR

## Abstract

We present a fluorometric method for determining ABC transporter activity in the pathogenic fungus *C. albicans* during different growth phases and in response to glucose. The carbocyanine dye diS-C_3_(3) was previously used to monitor plasma membrane potentials and test the influence of surface-active compounds in membrane polarization. We used diS-C_3_(3) to show changes in fluorescence kinetics that reflect changes in the activity of ABC transporters in *C. albicans* growth. Cdr1-GFP fluorescence, revealed that Cdr1p relocates to the inside of the cell after the early-log growth phase. Addition of glucose to the cell suspension resulted in Cdr1p transporter expression in the *CDR2*-knockout strain. We confirmed the diS-C_3_(3) results by standard RT-PCR and Western blotting.

## Introduction

*Candida albicans* normally occurs as a relatively harmless organism in the human microbiome (Koh, 2013); however, *C. albicans* infection can be triggered by various perturbations in homeostasis, such as compromised immune defense or breaks in the epithelium–blood barriers due to injury or surgery. Interestingly, the risk of infection is also increased in diabetic patients (Perlroth et al., 2007), possibly due to the dramatic effects of glucose on *C. albicans*’ metabolism (Brown et al., 2014) that increase virulence and drug resistance (Rodaki et al., 2009; Ene et al., 2012; Mandal et al., 2014). Moreover, Mandal et al. (2014) found that glucose selectively interacts with commonly used antifungal agents by forming complexes via hydrogen bonding, which, in turn, lowers their efficacy. The growing number of *C. albicans* strains resistant to pharmaceuticals is decreasing the already low number of drugs available to treat candidiasis. Due to *C. albicans’* multiple mechanisms to adapt to and resist drugs, new experimental approaches must be developed to define the *in vivo* and/or real-time behaviors of individual cells (Brown et al., 2014). Many of *C. albicans*’ adaptations and resistance mechanisms are related to the supramolecular structure formed by the cell wall and plasma membrane. Active transport through the plasma membrane is driven by transporters powered by high energy compounds, such as ATP, and/or membrane potential (Cannon et al., 2009). Thus, it is very important to develop methods to directly monitor drug transporters and plasma membrane-related activity in *C. albicans.*

*Candida albicans’* drug transporters were previously investigated via heterologous expression in *Saccharomyces cerevisiae*, but growing evidence indicates that *C. albicans’* metabolism is different from that of *S. cerevisiae*, especially in terms of major transcriptional modifications and resistance to osmotic stress and antifungal drugs (Garreau et al., 2000; Gasch et al., 2000; Ene et al., 2012; Szczepaniak et al., 2015). Glucose, for example, enhances oxidative stress resistance in *C. albicans* but decreases stress resistance in *S. cerevisiae* (Garreau et al., 2000; Gasch et al., 2000). Therefore, it is very important to account for many factors when looking for new, efficient treatment strategies of candidiasis.

We developed a fluorescence method that allows *in vivo* real-time monitoring of the activity of *C. albicans’* drug eﬄux pumps, Cdr1p, and Cdr2p, using a 3,3′-dipropylthiadicarbocyanine (diS-C_3_(3)) probe (Szczepaniak et al., 2015). The method is based on the property of diS-C_3_(3) to increase AAA_max_ after binding to cell constituents (mostly proteins); since the maximum fluorescence wavelength of the bound probe is about 10 nm higher than that of the free probe in solution, it allows us to observe its accumulation in cells and thereby monitor the actions of the probe-expelling pumps. This method also allows us to examine membrane potential differences in *S. cerevisiae* based on the changes of the fluorescence spectra of diS-C_3_(3) from equilibrium (Plášek et al., 2012). In this work, we used diS-C_3_(3) to assess the scope of *C. albicans* ABC transporter activity in response to membrane potential changes and glucose.

## Materials and Methods

### Strains and Growth Media

The *C. albicans* strains used in this study (**Table 1**) were generous gifts from D. Sanglard (Lausanne, Switzerland). All strains were grown at 28°C on YPD medium with 2% glucose, 1% Bacto peptone (Difco), and 1% yeast extract (Difco) with shaking at 120 rpm. Solid medium was supplemented with 2% agar.

**Table 1:** *Candida albicans* strains used in this study.

Strain	Genotype	Reference
CAF 2-1	*ura3Δ::imm434/URA3*	Fonzi and Irwin, 1993
DSY 448	*cdr1Δ::hisG-URA3-hisG/cdr1Δ::hisG*	Sanglard and Ischer, 1996
DSY 653	*cdr2Δ::hisG-URA3-hisG/cdr2Δ::hisG*	Sanglard et al., 1997
DSY 654	*cdr1Δ::hisG/cdr1Δ::hisG cdr2Δ::hisG-URA3-hisG/cdr2Δ::hisG*	Sanglard et al., 1997
DSY 4-2	*ura3Δ::imm434/ura3Δ::imm434*	Fonzi and Irwin, 1993
ASCa1	*ura3Δ::imm434/URA3 CDR1-GFP*	This study

### Strain Construction

Strain ASCa1 was constructed by integration of the *CDR1-GFP-URA3* cassette into the chromosomal locus of *CDR1* in the CAF 4-2 strain, as described by Gerami-Nejad et al. (2001). *CDR1*-specific sequences were added to universal primers to generate primers AS001 and AS003. We used primers AS003 and AS004 to verify integration into the chromosomal locus of *CDR1* (**Table 2**).

**Table 2:** Primers used in this study.

Primer name	Sequence	Reference
AS001	CATTCTTACGGTGATCTTTTATTGGTTAGCCAGAGAGAATAGAGTTCCAAAGGGTAAAAAAAATAAGAAAGGTGGTGGTTCTAAAGGTGAAGAATTATT	Larsen et al., 2006
AS002	ACAACAACAATAGTCTAAAAACGTCTATTATATTTTAGACGTTTGAGATACCACCATGTCAAAAAACAAATCTAGAAGGACCACCTTTGATTG	
AS003	ACATTAAATTTGCTGGTGGG	This study
AS004	CCTTCTGGCATGGCAGACTTG	
ACT1-F	TTTAAGAATTGATTTGGCT	Murzyn et al., 2010
ACT1-R	GAAGATTGAGAAGAAGTTT	
CDR1-F	TGCCAAACAATCCAACAA	Ricardo et al., 2009
CDR1-R	CGACGGATCACCTTTCATACGA	

### Sample Preparation

Cells were prepared according to the method of Gásková et al. (1998), with modifications. One hundred and fifty microliter of overnight, stationary culture were added to 20 ml of fresh YPD medium and incubated at 28°C with shaking at 120 rpm for 10, 14, or 24 h. The cells were then harvested by centrifuging at 110 × *g* for 3 min, washing twice with deionised water, and resuspending in citrate-phosphate (CP) buffer (pH 6.0) at OD_600_ = 0.1.

### DiS-C_3_(3) Uptake into Cells

Samples (3 ml, OD_600_ = 0.1) were labeled with diS-C_3_(3) at a final concentration of 5×10^-8^ M at room temperature. Fluorescence spectra were measured every 4 min for 120 min, with gentle stirring before each measurement, on a Fluorescence Spectrophotometer (HITACHI F-4500) equipped with a xenon lamp. The excitation wavelength was 531 nm and the fluorescence range was 560–590 nm. Scattered light was eliminated by an amber glass filter with a cut-off wavelength of 540 nm. If indicated, glucose was added at a final concentration of 2%.

### Microscopy Studies

Strains were grown for 24 h in YPD medium at 28°C with shaking at 120 rpm. At indicated times, aliquots of cell culture were pelleted by centrifuging, washed in deionised water, and 4 μl of samples were visualized with a ZEISS AXIO IMAGER.A2.

### Real-time PCR

The assay was prepared from samples (5 ml, OD_600_ = 0.4) after staining with 2×10^-7^ M diS-C_3_(3) probe for 40, 72, or 96 min, with 2% glucose added after 60 min if indicated. Aliquots of cell suspensions were pelleted by centrifuging at 2260 × *g* for 5 min. Cells were resuspended in lysis buffer (1 M sorbitol, 0.1 M EDTA, 1% β-mercaptoethanol, 2.5 mg/ml zymolyase), incubated at 37°C for 30 min, and centrifuged at 2834 × *g*. Total RNA was extracted using a Total RNA Mini kit (A&A Biotechnology) according to the manufacturer’s instructions. The purity and concentration of RNA samples were determined from A260/A280 readings and RNA integrity was checked by electrophoresis. Samples were treated with DNAse I (Fermentas) to remove genomic DNA contamination. The cDNA was synthesized using 0.5 μg RNA with a High-Capacity cDNA Reverse Transcription Kit (Applied Biosystems). Real-time PCR reactions with performed with a DyNAmo HS SYBR Green qPCR Kit (Thermo Scientific) and a 7500 Real-Time PCR System (Applied Biosystems). Gene-specific primers for actin (ACT1-F and ACT1-R) and *CDR1* (CDR1-F and CDR1-R) were used. The thermal cycling conditions consisted of the initial step at 50°C for 2 min, then 95°C for 10 min, followed by 35 cycles at 95°C for 20 s, 45°C for 20 s, and 72°C for 30 s. The gene expression level of the wild-type strain at 40 min of incubation, relative to that of the other time points, was calculated using the formula 2^-ΔΔCT^.

### Western Blotting

The assay was performed according to the method of Hiller et al. (2006), with modifications. Crude protein extract was prepared from samples (5 ml, OD_600_ = 0.4) after staining with 2×10^-7^ M diS-C_3_(3) probe for 10, 40, 72, or 96 min, with 2% glucose added after 60 min if indicated. Aliquots of cell suspensions were pelleted by centrifuging at 2260 × *g* for 5 min and resuspended in 1 ml of deionised water. Cells were lysed by the addition of 150 μl of 1.85 M NaOH-7.5% β-mercaptoethanol and incubated on ice for 10 min. Proteins were precipitated by the addition of 150 μl of 50% trichloroacetic acid and incubated on ice for 10 min. Samples were then centrifuged at 10,000 × *g* for 5 min at 4°C, washed in 1 ml of 1 M Tris-HCl (pH 8.0), and resuspended in 20 μl sample buffer (40 mM Tris-HCl, 8 M urea, 5% SDS, 0.1 mM EDTA, 1% β-mercaptoethanol, 0.1 mg/ml bromophenol blue), followed by incubation at 37°C for 30 min. Five microliter of protein extract were loaded in a 10% sodium dodecyl sulfate-polyacrylamide gel and electrophoresed in a Mini-PROTEAN II electrophoresis cell (Bio-Rad). After the electrophoresis, the samples were transferred onto a nitrocellulose membrane using a Mini-PROTEAN Tetra System electrophoresis cell (Bio-Rad). The membranes were stained with Ponceau S to check for equal loading of the gels. Immunodetection of Cdr1p was performed using a polyclonal rabbit anti-Cdr1p antiserum (a generous gift from D. Sanglard, Lausanne, Switzerland) and horseradish peroxidase-conjugated anti-rabbit antiserum as a secondary antibody. Signals were detected using an ECL kit from PerkinElmer according to the manufacturer’s instructions.

## Results and Discussion

### Membrane Potential, as Measured by diS-C_3_(3) Fluorescence, is a Factor in ABC Transporter Activity During Phases of *C. albicans* Growth

**FIGURE 1:**
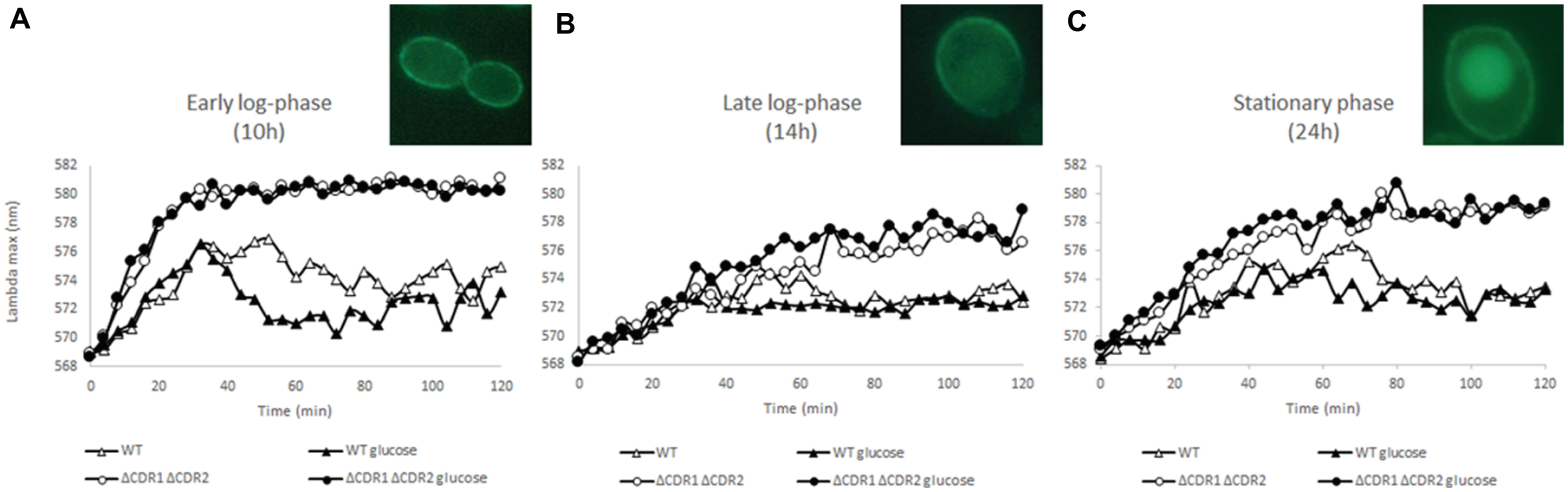
**DiS-C3(3) fluorescence staining of wild-type and CDR1Δ CDR2Δ strains during early log-phase **(A)**, late log-phase **(B)**, and stationary phase **(C)**.** 2% glucose was added at the start of the experiment (*n* = 3). Pictures: Cdr1-GFP localization during **(A–C)**.

As indicated by Plášek et al. (2012) and Plášek and Gášková (2013), diS-C_3_(3) is a suitable probe to monitor real-time changes in plasma membrane potential; here, we are the first to describe this use in *C. albi*c*ans*. To monitor the changes we measured the fluorescence AAA_max_ in the strain without ABC transporters to remove their effect on probe eﬄux. Probe accumulation took only 30 min in the early log-phase but took 60 and 50 min, respectively, to accumulate after 14 and 24 h in culture (**Figure 1**). As cultures age, the cells and the structure and function of their membranes change. The kinetics of diS-C_3_(3) fluorescence (**Figure 1**) reflect changes in the membrane potential of cells in different phases of growth. An age-induced reduction in membrane potential correlates with decreased polarization (Kumar et al., 2015). Cell membrane depolarisation can cause lateral redistribution of membrane proteins (Grossmann et al., 2007). Kumar et al. (2015) found that membrane fluidity contributes to drug diffusion and resistance. Higher membrane fluidity can lead to incorrect localization of the Cdr1p pump and thus lack of Cdr1p activity. The *C. albicans* strain with *CDR1* overexpression and drug resistance had a more rigid membrane than the drug-sensitive strain; this rigidity increased until the late-log phase of growth. On the other hand, the wild-type *C. albicans* SC5314 increased the fluidity of its membrane during growth. In the *C. albicans* mutants with deletion of a key component required during the biogenesis of mitochondria, decreases in the cellular ergosterol level and mis-sorting of Cdr1p into vacuoles were observed (Thomas et al., 2013). Similar effects were observed in the *S. cerevisiae* strain expressing *C. albicans CDR1* with deletions in the genes involved in ergosterol synthesis (Pasrija et al., 2008). Mukhopadhyay et al. (2004) found that Cdr1p dislocated from the cell membrane after incubating *Saccharomyces* cells with filipin, which interacts with the 3-hydroxyl group of membrane sterols. In this study, we observe that the Cdr1p pump fused with GFP is located in the plasma membrane in the early logarithmic phase (**Figure 1A**). Starting from the 14 h late log-phase, the movement of Cdr1p from the membrane to inside the cells becomes noticeable; strong fluorescence can be observed there in the stationary phase of growth (**Figures 1B,C**), although we do not see drop in pumps activity.

### Glucose Causes *de Novo* Synthesis of Cdr1p in CDR2Δ

**FIGURE 2:**
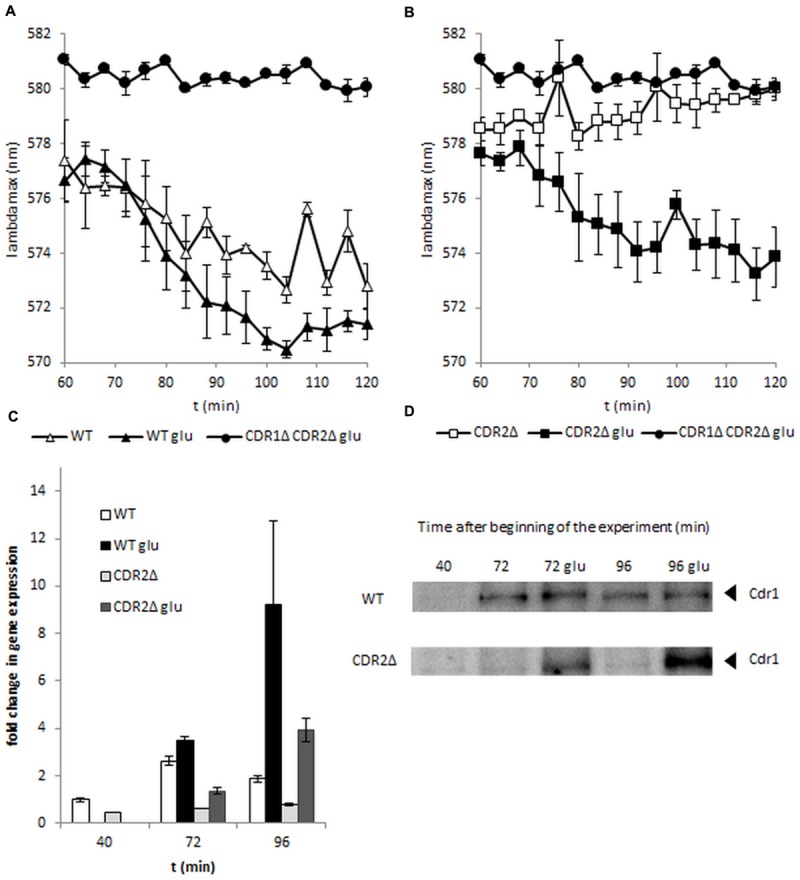
**DiS-C3(3) fluorescence staining of wild-type **(A)** and CDR2Δ **(B)** strains during early log-phase.** Glucose was added after 60 min of staining. **(C)** Expression of CDR1 during the experiment. Samples were taken at 40, 72, or 96 min from the beginning of the experiment. **(D)** Cdr1 protein levels during the experiment. Samples were taken at 40, 72, or 96 min from the beginning of the experiment (*n* = 3).

The most important factor in the activity of ABC transporters is ATP energy. *C. albicans* is a Crabtree-negative yeast and retains respiratory activity during growth at high glucose concentrations. Cdr1p and Cdr2p are up-regulated in the presence of glucose and this process probably increases resistance to azoles (Rodaki et al., 2009). To see if diS-C_3_(3) export can be facilitated by glucose we added it at the beginning of the experiment. In the early log-phase of growth (**Figure 1A**) glucose promotes dye eﬄux but this effect fades in further stages of culture (14 h late-log and 24 h stationary phase; **Figures 1B,C**). Interestingly the most marked change could be observed in *cdr2Δ* strain (**Figure 2B**). In the experiment without glucose *CDR1* pump was inactive (**Figure 2B**) and the difference in AAA_max_ between it and after the addition of glucose was more pronounced than in the strain with both pumps (**Figures 2A,B**). This fact indicates that Cdr2p actively participates in removing diS-C_3_(3) from the fungal cells.

We used standard molecular methods to assess if the increased activity of Cdr1 under the influence of glucose was due to pump activation or pump overexpression. As shown in **Figure 2D**, western blot showed high levels of Cdr1 protein after induction of glucose in a strain lacking Cdr2p, whereas Cdr1p was observed at very low levels in control conditions (**Figure 2D**). In contrast, in the wild-type strain, Cdr1p was detected after 72 min but the level of this protein appeared to be the same, regardless of whether glucose was added (**Figure 2D**); this was reflected in both strains’ diS-C_3_(3) eﬄux. As shown in **Figure 2C**, *cdr2Δ* expresses *CDR1* continuously at a very low level. Glucose addition in *cdr2Δ* causes a twofold increase in *CDR1* expression after 12 min and fourfold increase after 36 min. Interestingly, under the same conditions in the wild-type strain, *CDR1* transcript level change is not detectable at 12 min after addition of glucose; after 36 min we observe a fivefold increase in transcript which do not correlate with protein level at this time point. This delayed response of wild-type strain to glucose may be a natural lag in between the gene expression and protein levels. Although *cdr2Δ* faster reaction could be a result of this strain need to complement deleted *CDR2*. Both gene expression and Western blotting suggested that glucose caused *de novo* synthesis of the Cdr1 pump in *C. albicans cdr2Δ*.

ABC transporter upregulation in *C. albicans* can be caused by antifungal drugs (Henry et al., 1999; Coste et al., 2004; Schneider and Morschhäuser, 2015), antibiotics (Vogel et al., 2008), or human steroid hormones (Larsen et al., 2006; Banerjee et al., 2007). *C. albicans* is a human pathogen; environmental glucose may be another sign that yeast cells have entered the bloodstream and to adapt express a virulence phenotype. One of the virulence factors of *C. albicans*, its ability to form hyphal forms, can be triggered by addition of serum to cultures at 37°C (Hudson et al., 2004). The active component starting this process is glucose; changes in *C. albicans* transcriptome, including *CDR1* and *CDR2* (Rodaki et al., 2009), start as early as 30 min after the addition of no more than 0.01% glucose – a concentration much lower than that in human serum (0.06–0.1%). Our results using diS-C_3_(3) also suggest that activation of Cdr1p under the influence of glucose results in overexpression of this pump, especially in case where *CDR2* is deleted.

## Conclusion

In this study, we showed that the diS-C_3_(3) carbocyanine probe can be used as a multipurpose fluorescent assay.

• DiS-C_3_(3) can be used to assess both membrane polarization and ABC transporter activity in *C. albicans*.
• Increase of the export diS-C_3_(3) by glucose depends on growth phase and is the strongest at the beginning of the log-phase.
• In case of *cdr2Δ* strain observed increase in diS-C_3_(3) export is caused *de novo* synthesis of Cdr1p.

## Author Contributions

JS execution of experiments; ML conceptual work; AK conceptual work and and writing publication.

## Conflict of Interest Statement

The authors declare that the research was conducted in the absence of any commercial or financial relationships that could be construed as a potential conflict of interest.
